# Metabolic biomarkers of radiotherapy response in plasma and tissue of an IDH1 mutant astrocytoma mouse model

**DOI:** 10.3389/fonc.2022.979537

**Published:** 2022-10-24

**Authors:** Victor Ruiz-Rodado, Tyrone Dowdy, Adrian Lita, Tamalee Kramp, Meili Zhang, Dorela Shuboni-Mulligan, Christel Herold-Mende, Terri S. Armstrong, Mark R. Gilbert, Kevin Camphausen, Mioara Larion

**Affiliations:** ^1^ Neuro-Oncology Branch, Center for Cancer Research, National Cancer Institute, National Institutes of Health, Bethesda, MD, United States; ^2^ Radiation Oncology Branch, Center for Cancer Research, National Institutes of Health, Bethesda, MD, United States; ^3^ Division of Neurosurgical Research, Department of Neurosurgery, University Hospital Heidelberg, Heidelberg, Germany

**Keywords:** Astrocytoma, metabolomics, biomarker, radiotherapy, 13C-tracing

## Abstract

Astrocytomas are the most common subtype of brain tumors and no curative treatment exist. Longitudinal assessment of patients, usually *via* Magnetic Resonance Imaging (MRI), is crucial since tumor progression may occur earlier than clinical progression. MRI usually provides a means for monitoring the disease, but it only informs about the structural changes of the tumor, while molecular changes can occur as a treatment response without any MRI-visible change. Radiotherapy (RT) is routinely performed following surgery as part of the standard of care in astrocytomas, that can also include chemotherapy involving temozolomide. Monitoring the response to RT is a key factor for the management of patients. Herein, we provide plasma and tissue metabolic biomarkers of treatment response in a mouse model of astrocytoma that was subjected to radiotherapy. Plasma metabolic profiles acquired over time by Liquid Chromatography Mass Spectrometry (LC/MS) were subjected to multivariate empirical Bayes time-series analysis (MEBA) and Receiver Operating Characteristic (ROC) assessment including Random Forest as the classification strategy. These analyses revealed a variation of the plasma metabolome in those mice that underwent radiotherapy compared to controls; specifically, fumarate was the best discriminatory feature. Additionally, Nuclear Magnetic Resonance (NMR)-based ^13^C-tracing experiments were performed at end-point utilizing [U-^13^C]-Glutamine to investigate its fate in the tumor and contralateral tissues. Irradiated mice displayed lower levels of glycolytic metabolites (e.g. phosphoenolpyruvate) in tumor tissue, and a higher flux of glutamine towards succinate was observed in the radiation cohort. The plasma biomarkers provided herein could be validated in the clinic, thereby improving the assessment of brain tumor patients throughout radiotherapy. Moreover, the metabolic rewiring associated to radiotherapy in tumor tissue could lead to potential metabolic imaging approaches for monitoring treatment using blood draws.

## Introduction

Lower grade gliomas (LGGs) that harbor IDH1^mut^ are the less aggressive form of gliomas; however, malignant transformation towards a more aggressive phenotype occurs (1, 2). For example, astrocytomas harboring an IDH1 mutation progress towards a highly aggressive phenotype (3). These malignancies tend to spread into neighboring healthy tissue; thus, surgical interventions present limitations, which makes radiotherapy (RT) a critical component of the therapeutic approach to these tumors. Monitoring the response to RT and other treatments is generally performed through Magnetic Resonance Imaging (MRI) and symptomatic evaluation. While these approaches are the current standard of care, an earlier distinction between radiation-induced necrosis and tumor progression than is currently offered by MRI would be beneficial for patient treatment (1). Therefore, plasma-based biomarkers represent attractive alternatives to monitor treatment response. Plasma-based metabolomics has been utilized to provide markers of CNS (Central Nervous System) tumors in the past (4, 5), as well as a reporter of progression and response to treatment in mouse models of cancer and patients (6). Typically, Liquid Chromatography Mass Spectrometry (LC/MS) and Nuclear Magnetic Resonance (NMR) are the techniques utilized to profile the blood fraction of choice for analysis. Datasets arising therefrom are subsequently examined for biomarkers or trends that allow the classification of subjects according to a disease, treatment, etc. Metabolomics investigations can experience the deleterious effects arising from multicollinearity and overfitting due to the nature of the experiments, i.e. few subjects and hundreds of variables. Accordingly, data analysis strategies have been implemented to overcome related challenges in the metabolomic studies of bio-fluids (7, 8). Additionally, metabolic profiling of brain tissue can also serve as an in-situ reporter of RT response as it carries the metabolic signature derived from the treatment. Metabolic information contained in different blood fractions have been previously utilized in diagnosis of gliomas (9) and in investigations involving the response to radiotherapy in cancer (10, 11). However, the assessment of response to RT in an animal model of astrocytoma in both tissue and plasma have not been reported yet. Studies addressing the metabolic profiling of bio-samples for radiotherapy monitoring are still severely limited (12–14). Herein, we report the plasma metabolic profiling of a grade III astrocytoma mouse model undergoing radiotherapy in addition to providing a series of time-related biomarkers associated with treatment response. Furthermore, we dissected the metabolic pathways affected from the treatment through the LC/MS and NMR analysis of tumor tissue and ^13^C-tracing experiments utilizing uniformly labeled ^13^C Glutamine, [U-^13^C]-Glutamine. ^13^C flux from glutamine towards succinate was significantly increased in the treated cohort whilst total lactate levels were decreased, which may indicate a potential rewiring of the metabolism within the tumor due to radiotherapy.

## Materials and methods

### Animal work

NCH1681 cell line was originated from an IDH1 mutant grade III astrocytoma patient (33 years old) (15). The treatment of primary tumor involved temozolomide and treatment of progressive disease by proton therapy before surgery of first recurrence. NCH1681 cells were maintained in DMEM:F12 Glutamax, supplemented with EGF, FGF, antibiotics and BIT (PeloBiotech, Martinsried, Germany) (16). Once cells reached the required number in culture, they were harvested, washed with phosphate buffered saline (PBS) and counted. The resulting pellets were resuspended in Hank’s Balanced Salt Solution, and 5 μL of this cell suspension (500,000 cells/mouse) were injected stereotactically into the striatum of 6–8 weeks old female SCID (severe combined immunodeficient) mice (Charles River Frederick Research Model Facility) using a stereotactic device. The intracranial orthotopic mouse model with the IDH1 mutant glioma cell line NCH1681 was established according to approved animal study proposal NOB-008 by the National Cancer Institute−Animal Use and Care Committee. Tumor growth was monitored for neurological symptoms daily. To determine endpoint, an independent researcher performed health assessment of the mice twice a day without previous knowledge of the experiment in course. Once this researcher determined that a mouse was reaching end point in view of previously defined symptoms, that mouse was euthanized. Symptoms include animal experiencing rapid weight loss (>15%, monitored daily), debilitating diarrhea, rough hair coat, hunched posture, labored breathing, lethargy, persistent recumbence, significantly abnormal neurological signs, bleeding from any orifice, self-induced trauma, impaired mobility, becomes moribund or otherwise becomes unable to obtain food or water. For comparison of survival curves, the log-rank (Mantel–Cox) test has been used (GraphPad Prism 7).

To examine the effects of radiation in control mice, we used 5 C57BL/6 mice obtained from Charles River Laboratories (CRL, Dublin, VA). Mice were sampled across 7 time points, one day before radiation and 6 timepoints after radiation (3 hrs, 6 hrs, 1 day, 4 days, 12 days and 24 days). Blood samples were taken 2 hrs before lights off for all time points except 6 hrs-post radiation which was sampled 2hr after light off. Micro sample tubes coated with lithium heparin (Sarstedt AG & Co., Germany) were used to collect 100 uL of blood from the mandibular vein, samples were centrifuged at 3,500 g for 15 min at 4°C to collect plasma and flash frozen with dry ice then stored at -80°C until analysis. Radiation was given using a small animal Pentax x-ray irradiator to anesthetized mice (ketamine: 80–120 mg/kg and xylazine: 5–25 mg/kg) restrained in a lead shielded apparatus designed to isolate radiation to only the brain. Mice were allowed to recover from anesthesia on heated pads for a maximum of two hours and returned to home cages after recovery.

### Animal radiotherapy

Radiation was performed on mice intracranially injected with the NCH1681 cell line. 30 days after injection, the mice were randomized in two groups, one undergoing RT (n=9 mice) and the other one as control (n=9 mice). Mice were irradiated with a total of 12 Gy; specifically, animals were treated on Monday and Friday for 2 consecutive weeks at 3Gy/session. Radiation was performed in a Pantek machine an orthovoltage radiotherapy unit. The mice were anesthetized with a cocktail of ketamine/rompun/saline mixture, i.e. ketamine (100 mg/ml), rompun (20 mg/ml) and diluted with saline to give the mice a 100 mg/kg dose of ketamine and 10 mg/kg rompun. The mice were injected with the cocktail at a dose of 0.01 µL per gram of half of the mouse’s body weight. Animals were then placed in a custom-made jig that only exposes the mouse brain to radiation while sheilding the body, including the eyes, ears, the oral cavity, and the spinal cord. The non-irradiated control mice were administrated both anesthesia and atipamezole. The radiated mice were observed for how much time they would have to be sedated for radiation and how long time passed until they received atipamezole, approximately. Those same parameters were used on the non-irradiated control mice. After radiation, the mice were given atipamezole, a reversal agent, to aid in the recovery.

### ^13^C tracing *in vivo*

When mice reached endpoint, they were injected with [U-^13^C]-glutamine and tumor was harvested for both quantification of ^13^C incorporation by NMR and metabolic profiling through LC/MS. [U-^13^C]-glutamine was injected to mice reaching endpoint at similar time points to improve consistency, i.e. mice undergoing radiotherapy utilized for ^13^C tracing analysis have an average survival of 92 days and control animal 82 days. Injections were performed as previously described (17, 18); briefly, [U-^13^C]-glutamine (Cambridge Isotopes) was prepared as a 36.2 mg/ml stock solution in sterile PBS and injected (200 μL, 7.24 mg) through the tail vein at 15 min intervals for 3 times (total = 142 μmol) just prior to mice reaching endpoint. Mice were euthanized 15 min after the last injection (45 min from the first injection). Tumors were separated from the brain and both tissues were gently blotted and flash-frozen in liquid nitrogen.

### Plasma processing for metabolic profiling

Blood was collected approximately every 10 days from the tail vein of the mice in Li-heparin collection tubes; subsequently, the sample was separated into plasma and packed cells by centrifugation at 3,500 g for 15 min at 4°C and stored at -80°C until extraction. 35 µL of plasma were extracted in a 1:2:1 water:methanol:chloroform mixture. Centrifuged for 20 min at 4°C and 13,000 rpm and the resulting upper hydrophilic phase was then transferred to a clean vial and dried under a stream of N_2_ gas. Dried sediments were resuspended in 60% methanol (aq.) and injected into the LC/MS system for global profiling. Blood samples were collected from all the mice at each time point, although 5 mice were selected for each group for biomarker discovery in order to account for blood samples at all the time points for the same animal, since mice deceased over time.

### Tissue processing for metabolomics

When mice reached endpoint, malignant tissue and contralateral regions were collected. Tumor tissue was first weighted as frozen for metabolite quantification purposes and to normalize the metabolite levels computed by LC/MS; subsequently, the sample was stored at -80°C for further processing. Tissue samples were mechanically lysed utilizing a bullet blender, and metabolite extraction was performed in a 1:2:1 water:methanol:chloroform solution. Then, samples were centrifuged at 12,000 rpm, for 20 min. at 4°C. The two resulting phases (upper aqueous polar and lower organic lipid) were separated, and the polar one was split in 2 (for NMR and LC/MS analyses) and dried under a stream of N_2_. Samples were resuspended in methanol for LC/MS analysis or in 180 μL of pH 7 phosphate buffer (100 mM) in D_2_O (containing d-TSP) and 0.1% NaN_3_ for NMR experiments. These tissue extracts were utilized for both quantification of ^13^C incorporation into metabolites *via* NMR and metabolic profiling by LC/MS.

### NMR acquisition and processing

NMR spectra were acquired on a 700 MHz Bruker Avance Neo (US National Cancer Institute, Bethesda, US). For 1D ^1^H experiments we utilized the noesygppr1d pulse sequence for water suppression involving 64 scans with a relaxation delay of 3 s, a spectral width of 12,000 Hz and 32,000 data points. Spectra were zero-filled to 64K points and we applied an exponential line broadening function of 0.3 Hz. 1D HSQC experiments for ^13^C tracing were acquired using the hsqcetgpsisp2.2 pulse sequence over 400 scans, 3,500 data points and a spectral width of 8,200 Hz. We applied an exponential line broadening function of 4 Hz and a Gaussian function of 7.5 Hz. All data were referenced to the TSP internal standard signal (s, δ = 0.00 ppm), phased and baseline corrected using ACD Labs Spectrus Processor 2016. For quantification, data was normalized to the TSP singlet and tissue weight and corrected for natural abundance of ^13^C (1.1%). Assignment of metabolites was done on the basis of literature values (19, 20) and available databases (21). The formula utilized to compute the nmol/mg of tissue from a 1d-hsqc is:

$C=\;\frac{A}{A_{TSP}}\;x\;\frac{nH_{TSP}^{+}}{nH^{+}}\;x\;C_{TSP}x\;\frac{V}{m}$ where: A are the areas under the peak of interest, nH^+^ are the number of protons attributable to each resonance signal, C_TSP_ is concentration of the TSP reference corrected for the natural abundance of ^13^C (1.11%), V is the volume of the sample and m the tumor mass.

### LC/MS global profiling of plasma and tissue

LC/MS analysis was conducted with the Agilent 6545 QTOF-MS combined with 1290 Infinity II UHPLC system (Agilent Technologies, Wilmington, DE, USA). Only LC/MS grade solvents and additives purchased from Covachem (CovaChem, LLC., Loves Park, IL, USA) were used to prepare mobile phases and wash solutions. Wash cycles consisting of strong wash (50% Methanol, 25% Isopropanol, and 25% Water), weak wash (90% Acetonitrile and 10% Water), and seal wash (10% Isopropanol and 90% water) were implemented to eliminate carryover between injections. Dried extracts were reconstituted in 80 µL 60:40 MeOH/H_2_O and samples were injected (8 µL) to resolve analytes using Infinity 1290 in-line filter combined with AdvanceBio Glycan Map 2.1 x 100mm, 2.7µm column (Agilent Technologies, Wilmington, DE., USA) set at 35^0^C. The solvent buffers were composed of mobile phase A (10 mM ammonium acetate in 88% water/12% acetonitrile) and mobile phase B (10 mM ammonium acetate in 90% Acetonitrile) titrated with formic acid and ammonium hydroxide to pH 6.85. The linear gradient was executed at flow rate 0.2 mL/min, as follows: 100% B, 0.5 min; 95% B, 2.0 min; 60% B, 3.0 min; 35% B, 5 min; hold 0.25 min; 0% B, 6 min; hold 0.5 min; 100% B, 7.8 min; equilibrate for 1.7 min. The mass analyzer acquisition parameters include drying gas temperature, 250^0^C; drying gas flow, 9 L/min; sheath gas temperature, 325^0^C; sheath gas flow, 11 L/min; nebulizer, 45 psig. Mass spectra were acquired at 3.0 spectra/s in negative electrospray ionization (ESI-) mode for a mass range from 72 to 1200 m/z using a voltage gradient of capillary 3000 V, nozzle 2000 V, fragmentor 80 V, skimmer 50 V, and octopole radio frequency 750 V.

### LC/MS data analysis

Prior to preprocessing datasets, pooled QC samples were inspected for consistency of retention time shifts and signal degradation. Following acquisition, m/z spectra binning was performed by partitioning the m/z vs retention time (RT) matrices into fixed width using Agilent Masshunter Profinder B.08.00. Bins were manually inspected to confirm consistent integration for all analytes detected across all samples. Targeted TOF-MS extraction of precursor ions was performed using in-house compound library. Ion selection and alignment parameters were restricted to proton loss (H-) in ESI-, H+ gain in ESI+, 5.0 mDa mass range, and retention time span ± 0.4 min. Following pre-processing, the areas for each analyte from each sample was corrected by area of sample-specific internal standard, p-nitrobenzoate and debrisoquine. Same acquisition procedure was followed for the ^13^C isotopically labeled samples. After alignment and identification of analytes of interest retention times a PCDL card was constructed using PCDL Manager B.07.00 (Agilent). The chromatograms were introduced in Agilent MassHunter Profinder B.08.00 and the PCDL card was used in the Batch Isotopologue Extraction routine with the following parameters: 99% ^13^C labeling, 20% peak height ion abundance criteria, mass tolerance of ± 15ppm +2 mDa with a threshold of 250 counts for anchor and 1000 counts for the sum of ion heights with a minimum correlation coefficient bigger then 0.5. Total levels of metabolites of interest included both the unlabeled and all the labeled isotopologues.

### Statistical analysis

MetaboAnalyst 4.0 (22) was employed for multivariate analysis including MEBA (multivariate empirical Bayes time-series analysis) (23) and multivariate ROC (receiver operating characteristic) curve analysis. MEBA was utilized to select the metabolites according to their correlation with the treatment over time. Top 15 ranked metabolites were evaluated as biomarkers by ROC curve analysis in a multivariate fashion that involved Random Forest as a classification strategy. The area under the curve (AUC) was the measure of separability as a function of treatment. GraphPad Prism 7 was employed to perform ANOVA for repeated measures and outliers were removed if >5 times the standard deviation.

## Results

### Radiotherapy for a mouse model of IDH1 mutant glioma

**Figure 1:**
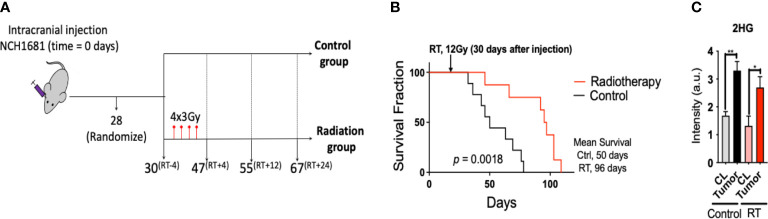
Radiotherapy for a mouse model of IDH1 mutant astrocytoma: **(A)**, Diagram of the experimental set up of the investigation (superscripts indicating the time in days relative to radiotherapy). **(B)**, Survival curves for the glioma mouse model under radiation and control (RT, radiotherapy) (n=9). **(C)**, 2HG levels obtained by LC/MS analysis in the tissues indicated (n=4 samples/group). Statistical significance from a two way-ANOVA followed by Sidak’s multiple comparison test displayed as **, p<0.005; *, p<0.05 (only significant differences are indicated).

Radiotherapy was applied to the intracranial IDH1 mutant glioma model in 2 consecutive weeks at 3 Gy per dose, 2 days per week (**Figure 1A**). Cox-Mantel test was utilized to assess the efficacy of the treatment delivering a significant p-value of 0.0018 and median survival values of 50 and 96 days for the control and RT groups respectively (**Figure 1B**). These results reveal a beneficial effect of RT on survival for our glioma mouse model. We also evaluated if the presence of IDH1 mutation in this model was translated to 2HG formation *in vivo*. 2HG levels were assessed by LC/MS in the different tissues, i.e. contralateral (CL) and tumor, revealing the expected higher levels of 2HG in the malignant tissue compared to the CL region in both treated and untreated mice (**Figure 1C**). In addition, we validated the presence of IDH1 in NCH1681 using DNA sequencing, DNA methylation profiling, and Western Blot analysis of IDH1 mutant protein (**Figure S3**).

### Plasma biomarkers of radiation in a glioma mouse model

**Figure 2:**
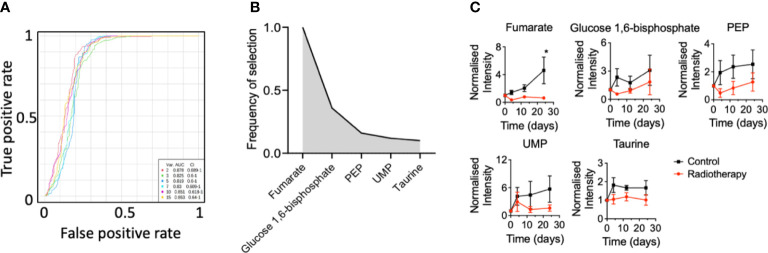
Plasma biomarkers of radiation for an astrocytoma mouse model: **(A)**, Multivariate ROC curve diagram for those metabolites selected by MEBA with the highest potential as plasma biomarkers based on the area under the curve. **(B)**, Top-5 ranked variables included in the multivariate ROC curve analysis and **(C)** their levels over time (n=5 mice per group, metabolites levels are displayed as mean ± SEM, significance assessed by a two-way ANOVA for repeated measures followed by Sidak’s test for multiple comparisons. *, p<0.05).

Since the NCH1681 model responded positively to radiotherapy, we conducted a plasma metabolomics investigation to evaluate the effect of RT in the plasma metabolome over time in 5 mice per cohort. Plasma metabolomics can provide a snapshot of the overall metabolic status of an organism and reveal potential metabolic biomarkers of RT response. The importance of the temporal changes of metabolites was assessed by MEBA, which is based on multivariate empirical Bayes statistic (23). The Hotelling’s T^2^ parameter arising from this test was employed to select the top-15 metabolites which displayed differential levels over time (**Table S1**). These top 15 features were further assessed as plasma markers of radiation through multivariate ROC curve analysis (**Figure 2A**). The best performance was obtained with RF models including only 2 metabolites (AUC=0.878, CI=0.689-1) and the addition of more features did not improve the outcome of the classifier. Fumarate, glucose 1,6 biphosphate, PEP, UMP and taurine were those metabolites most frequently included in the models for samples classification (**Figure 2B**); although only fumarate levels attained statistical significance (from the multiple comparison test) at the last time point (**Figure 2C**), i.e. 24 days after last dose of radiation, similarly to glutamate (**Figure S1**). As a control, we irradiated normal mice and collected plasma pre-treatment 3 hours, 6 hours, 1 day, 4 days, 12 days and 24 days post-RT. Interestingly, fumarate levels did not change significantly in normal mice that underwent radiotherapy (**Figure S2**).

Two-way ANOVA revealed that glutamate, PEP and fumarate levels changed significantly between both cohorts (**Table S1**) and glutamate, dihydroxyacetone phosphate (DHAP), inosine and dUMP levels displayed a significant correlation with the factor ‘time’ (**Table S1**).

### Radiotherapy modifies the metabolic profile of glioma tissue

**Figure 3:**
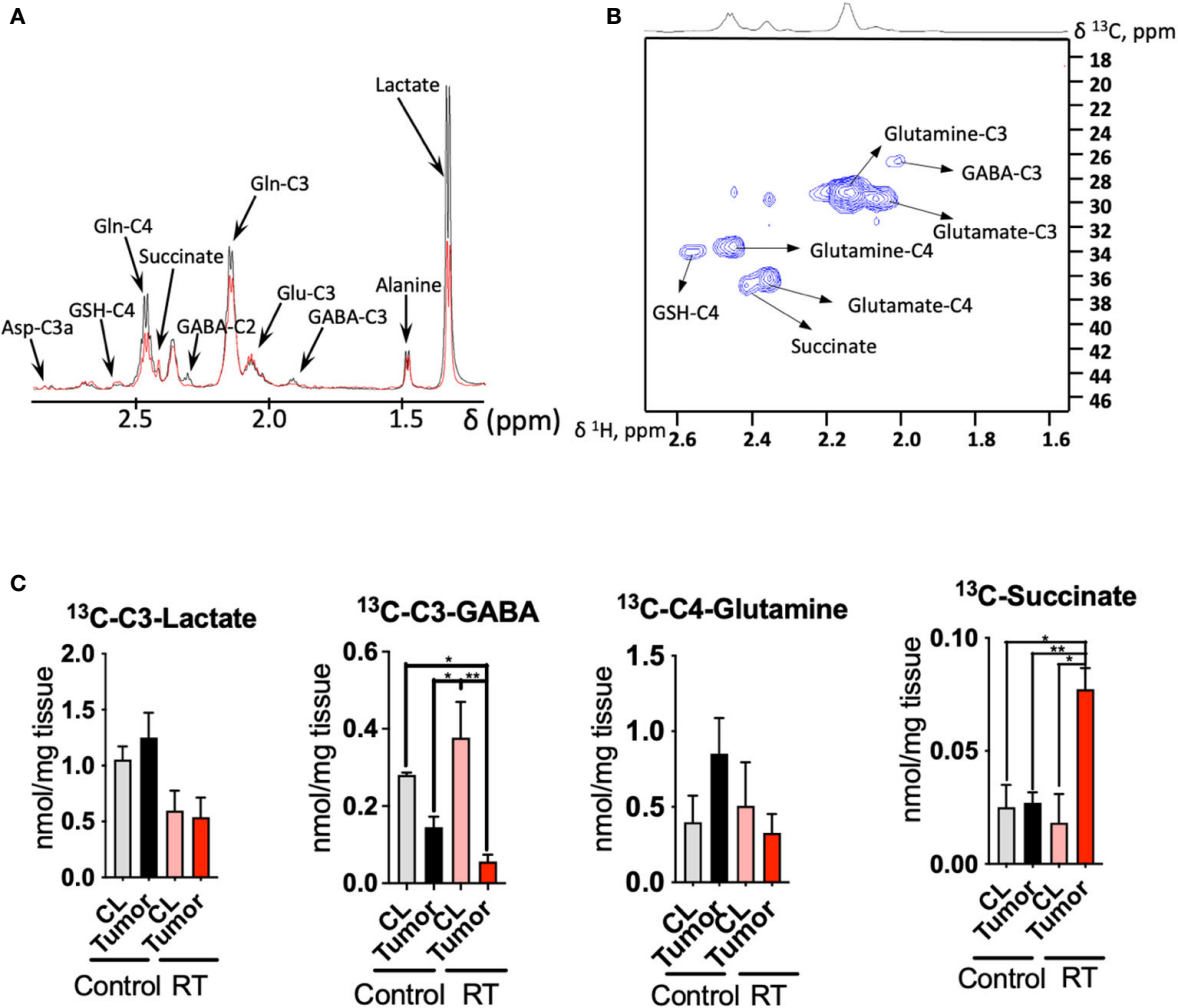
Radiotherapy enhances the 13C incorporation of glutamine to succinate in astrocytoma tissue: **(A)**, 1D HSQC NMR stack plot of spectra acquired on tumor tissue from a mouse from the control group (black) and undergoing radiotherapy (RT, red) displaying the resonances arising from ^13^C labeling from [U-^13^C]-glutamine tracing experiments. Typical spectra are shown (resonance intensities normalized to tumor weight). **(B)**, 2D ^1^H-^13^C HSQC Representative spectrum of a tumor sample from a mouse infused with [U-^13^C]-glutamine displaying the region between 1.6-2.6 ppm (^1^H) and 18-46 ppm (^13^C). **(C)**, Quantification of the [U-^13^C]-glutamine labeling on those metabolites showing a different degree of ^13^C incorporation after radiation in tumor and contralateral regions. (data displayed as bar plots ± SEM, n=3, statistical significance assessed through a two-way ANOVA followed by Tukey HSD test, *, p<0.05; **, p<0.005). Only significant differences are indicated. RT was 12Gy.

In order to explore the metabolic signature of radiotherapy within the tumor, we analyzed malignant tissue collected from both groups (treated and untreated) at end point by LC/MS (**Figure S4**) and NMR (**Figure 3**). From the 88 metabolites detected by LC/MS in profiling experiment, we observed a dysregulation of the glycolytic pathway in view of the number of metabolites associated to this metabolic route that were affected after radiation (**Figure S4A**). Levels of phosphoenolpyruvate (PEP) and glyceraldehyde-3P, both glycolytic intermediates, were downregulated in addition to lactate; contrarily, 1,3-biphosphoglycerate levels were higher for the radiation group. Hence, glucose metabolism was highlighted as a key metabolic pathway affected from treatment (**Figure S4B**). Energetic metabolites such as ATP and GTP were also upregulated in the treated cohort as well as further intermediates such as UDP and GDP; however, deoxy nucleotides including dUMP and dTMP were both downregulated in the radiation group. Indeed, pathway analysis (**Figure S4B**) revealed pyrimidine metabolism as one of the main pathways affected from radiation.

Additionally, we performed ^13^C tracing experiments by bolus injection of [U-^13^C]-glutamine through the mouse tail vein, since this amino acid has been revealed as a key metabolite in gliomas metabolism and contributes to fuel the TCA cycle, especially under hypoxic conditions (24–26) (**Figure 3**). Indeed, the utilization of inhibitors of glutaminase, the enzyme that converts glutamine to glutamate, has been proposed as a potential target in tumors that harbor the IDH1 mutant gene (27, 28). NMR analysis of the tissue extracts obtained from treated and control mice allow to quantify the incorporation of ^13^C units derived from [U-^13^C]-glutamine into downstream metabolites including lactate, alanine, GABA, glutamate, GSH and aspartate (**Figures 3A-C**). We observed downregulation of lactate production from ^13^C glutamine in the RT-treated cohort (p<0.05 for ‘treatment’ factor from two-way ANOVA), although these changes did not attain statistical significance for individual comparisons **Figure 3C**).

Tumor tissue displays a higher uptake of [U-^13^C]-glutamine in the control group, although the differences found through the ^13^C tracing experiment did not attain statistical significance. Previous investigations have reported ^18^F-Glutamine as an imaging marker of glioma (29) in view of its higher uptake by tumor tissue. Under RT, CL region displays similar levels to those encountered in the control mice. Lower levels of glutamine were found in tumor tissue compared to the CL region in those mice that underwent RT as well as the tumor from the control cohort. GABA synthesis was also downregulated in the tumor compared to the CL region for both groups (p<0.05 for ‘tissue’ factor CL vs tumor from the two-way ANOVA), and this effect was more intense under RT as glutamine is a major precursor for this neurotransmitter (30). The flux from [U-^13^C]-glutamine to succinate, a TCA cycle intermediate, was upregulated in the treated cohort (both interaction, ‘tissue x treatment’, and ‘tissue’ factors attained a p<0.05 from the two-way ANOVA); however, ^13^C succinate levels were similar to both CL regions and tumor tissue within the control group.

## Discussion

Radiotherapy is known to generate DNA damage and compromise its repair in addition to triggering radiolysis of cellular water that can originate free radicals that may cause chemical modifications in the DNA, proteins, and tumor microenvironment (31–33).

Tumors harboring a mutation in the IDH1 gene have been reported to present an enhanced sensitivity to radiation due to the modification of the epigenetic landscape by 2HG that affects the DNA damage responses (34). In addition, *de novo* pyrimidine pathway which contributes precursors to DNA synthesis has been recently reported to be a vulnerability of IDH1-mutated astrocytoma (35). The RT vulnerability has been exploited in IDH1-mutant tumors revealing a beneficial effect in response to radiation (36), as observed in our model as well (**Figure 1B**).

Interestingly, a recent investigation including a longitudinal study of brain tumor patients harboring the IDH1 mutation that received RT reported how 2HG levels decreased over time in oligodendroglioma patients under treatment. Astrocytoma patients experienced a comparable trend, although it was less pronounced (37). In our study, 2HG levels in tumors experienced a slight (non-significant) decrease in the treated cohort compared to the non-treated group (**Figure 1C**, black bars versus red bars). Together, these studies suggest the need to identify other biomarkers that could report on RT response.

Herein, we report biomarkers associated with RT in plasma of mice harboring an IDH1-mutated astrocytoma and which experienced a significant increase in survival as a result of RT. We discovered that glutamate and fumarate, were top-ranked by MEBA analysis, therefore, serving as biomarkers of RT. These metabolites are closely related since fumarate is one of the TCA cycle intermediates for which one of the entries involves the deamination of glutamate that produces α-ketoglutarate. Glutamate has been reported as downregulated in serum collected from GBM patients under RT (38). A recent investigation including human plasma from brain tumor patients and healthy individuals also highlighted the TCA cycle as a major dysregulated pathway in gliomas (39). Interestingly, a urinary metabolomics investigation including non-human primates subjected to radiation found fumarate to be downregulated in the urine collected from treated animals (40).

Next, we undertook a steady state analysis of tumor tissue. We observed that pyrimidine metabolism was significantly affected from RT (**Figure S4B**). This pathway is mainly involved in the synthesis of nucleic acids required for cell division and further proliferation. In fact, dTMP levels were also lower in tumor tissue collected from mice that underwent radiotherapy. This metabolite is a major metabolic component of proliferative processes including DNA synthesis. Therefore, reduced levels of dTMP may indicate reduced proliferation attributable to radiation, which may be reflected in the increased survival of the cohort undergoing RT. These results are interesting since *de novo* pyrimidine synthesis has been recently reported as a novel vulnerability in the IDH1-mutant astrocytoma models and suggests the potential for combining RT with *de novo* pyrimidine synthesis inhibitors (35).

Since the glutamate and fumarate were altered in plasma, we further looked at the [U-^13^C] glutamine incorporation into various tissue metabolites. Interestingly, ^13^C labeling of succinate was upregulated, reflecting an increased flux from the [U-^13^C] glutamine. Since succinate is part of many pathways we speculated where succinate might be needed. Succinate can be produced within the nucleus through TET enzymes, although this process is known to be inhibited by 2HG, that acts as a competitive inhibitor of α-ketoglutarate-dependent dioxygenases (41). Notably, GABA is a precursor of succinate through the activities of GABA transaminase and succinic semialdehyde dehydrogenase (SSADH). Therefore, the decrease in ^13^C-labeled GABA in tumor tissue under RT along with the significant increase in ^13^C-succinate levels may indicate a higher flux of GABA towards succinate as a response to treatment. Interestingly, increased succinic semialdehyde dehydrogenase (SSADH) levels, the enzyme that converts GABA into succinate were found in highly proliferative areas within the glioblastoma (42). This is one hypothesis, however, the fate of ^13^C labeled succinate is not very clear, since we were not able to detect fumarate ^13^C peaks with our NMR approach.

Taking all these changes together, a dysregulated glutamine metabolism in tumor tissue is observed as a consequence of RT treatment. The increased levels of ATP and GTP in the radiation group can be attributed to an increased transference of energy carriers from the microenvironment in order to maintain the proliferation levels despite the deleterious effects of RT (43). However, we have also observed an increase in the total levels of oxoglutarate and a higher flux from [U-^13^C]-glutamine to succinate which may indicate an upregulation of the succinyl CoA synthase which yields GTP; intriguingly, the following metabolites, such as fumarate and oxaloacetate, within the TCA cycle, display lower levels in the radiation group which may indicate downregulation of succinate dehydrogenase due to RT.

We have previously reported that the IDH1-mutant mouse model of NCH1681 exhibits an active glycolytic pathway that yields lactate *in vivo* (1), similarly to other aggressive IDH1 mutant glioma models (44). This observation indicates the utilization of this metabolic route to meet the high energetic demands for the enhanced proliferation characteristic of tumors. Lower levels of lactate reported in this investigation in the radiation group may serve as additional evidence for this metabolite to be employed as a marker of RT response in the gliomas (45). Indeed, a recent investigation revealed a decreased glycolytic activity in radiation-induced necrotic tissue based on lower lactate formation in brain tumors and metastasis including CL regions of irradiated mice as controls (46). Additionally, our ^13^C tracing experiment revealed a significant increase in glutamine-derived succinate levels in tumor tissue after RT and not in the CL of mice from the same cohort. This metabolic response may highlight a potential marker of specific metabolic rewiring in gliomas subjected to radiation. However, the results presented herein are obtained by utilizing one animal model; therefore, preclinical and clinical validation including other glioma models and plasma obtained from patients must be conducted.

## Conclusions

Herein, we have reported how plasma metabolomics could be employed as an alternative strategy for assessing response to radiotherapy in glioma. We observed significant changes in the plasma metabolic profile throughout the course of the mice lifespan after treatment which could be validated in the clinic in human patients. Additionally, we describe the radiation-induced changes in tumor tissue, and more specifically, those attributable to the utilization of glutamine which can be further explored by molecular imaging approaches in clinical settings.

## Data availability statement

The LC-MS plasma metabolomic dataset generated is available at the NIH Common Fund’s National Metabolomics Data Repository (NMDR) website, the Metabolomics Workbench, https://www.metabolomicsworkbench.org where it has been assigned Project ID PR001096. The data can be accessed directly *via* it’s Project DOI: 10.21228/M81M6P.

## Ethics statement

The animal study was reviewed and approved by Approved animal study proposal NOB-008 by the National Cancer Institute−Animal Use and Care Committee.

## Author contributions

VR-R and ML: Conceptualization. ML: Project administration, supervision and funding acquisition. TD, AL, TK, MZ: Formal analysis. CH-M, KC, MG, and ML: Resources. VR-R: Writing—original draft preparation. VR-R, TD, AL, TK, MZ: Formal analysis. TD, AL, TK, MZ, CH-M, KC, TA, DS-M, MG, and ML: Writing—review and editing. All authors have read and agreed to the published version of the manuscript All authors contributed to the article and approved the submitted version.

## Funding

This work was supported by the Intramural Research Program, Center for Cancer Research, National Cancer Institute, National Institutes of Health. This work is supported by NIH grant U2C-DK119886.

## Acknowledgments

We would like to thank Hua Song, Dionne Davis and Wei Zhang for their help with the animal work.

## Conflict of interest

The authors declare that the research was conducted in the absence of any commercial or financial relationships that could be construed as a potential conflict of interest.

## Publisher’s note

All claims expressed in this article are solely those of the authors and do not necessarily represent those of their affiliated organizations, or those of the publisher, the editors and the reviewers. Any product that may be evaluated in this article, or claim that may be made by its manufacturer, is not guaranteed or endorsed by the publisher.
