# Supplementary material for: Metabolic biomarkers of radiotherapy response in plasma and tissue of an IDH1 mutant astrocytoma mouse model
Source: Front Oncol. 2022 Oct 24;12:979537. doi: 10.3389/fonc.2022.979537 (PMC9638021; doi:10.3389/fonc.2022.979537)

## Supplementary Figures and Tables

**Supplementary Table S1.** Top-15 ranked metabolites by MEBA: Table displaying the top 15 features highlighted by MEBA including the pathways and superpathways they belong to, and the p-values arising from a two-way ANOVA for repeated measures including 2 factors (treatment and time). Abbreviations: 2-PG, 2-Phosphoglycerate; 3-PG, 3-Phosphoglycerate; PEP, Phosphoenolpyruvate; DHAP, Dihydroxyacetone phosphate; ns, not significant. None of the features attained statistical significance for the interaction factor (treatment x time).

| Metabolite | p (Treatment) | p (Time) |
|---|---|---|
| NAD | ns | ns |
| 4-Hydroxy-2-ketoglutarate | ns | ns |
| Glutamate | 0.027 | 0.0017 |
| Taurine | ns | ns |
| 2-PG | ns | ns |
| 3-PG | ns | ns |
| Glucose 1,6-bisphosphate | ns | ns |
| PEP | 0.049 | ns |
| DHAP | ns | <0.0001 |
| Malate | ns | ns |
| Fumarate | 0.035 | ns |
| $\alpha$-Ketoglutarate | ns | ns |
| Inosine | ns | 0.0474 |
| dUMP | ns | 0.019 |
| UMP | ns | ns |

**Supplementary Figure S1: Top ranked metabolites from MEBA:** Metabolites levels over time are displayed as mean ± SEM (n=4-5 mice per group, significance assessed by a two-way ANOVA for repeated measures followed by Sidak's test for multiple comparisons. *, p<0.05).

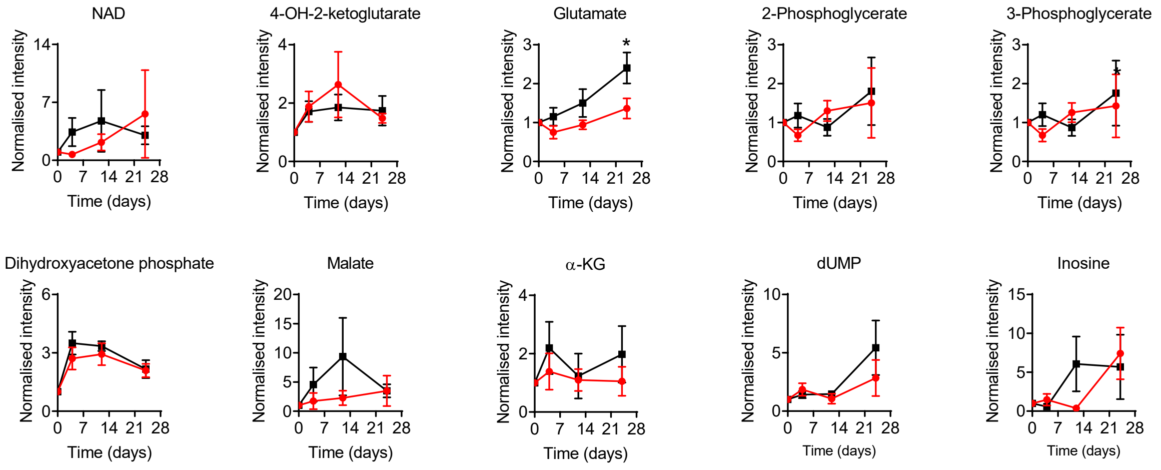


**Supplementary Figure S2**: **Fumarate levels in normal mice as a result o RT.** Fumarate levels over time are displayed as mean ± SEM (n=4-5 mice per group, significance assessed by a two-way ANOVA. *, p<0.05). None of the time points attain significance when compared with the levels before RT.

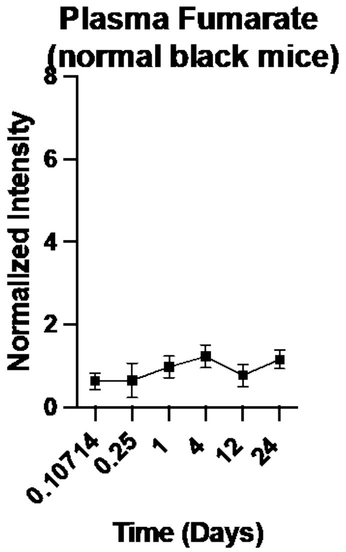

**Supplementary Figure S3**: **Validation of IDH1 mutation in NCH1681 cells**. A. DNA methylation-based tumor classification and copy number variation plot of glioma cell lines including the classification scores arising from their methylome profile. NCH1681 cell lines were matched to high-grade IDH$^{mut}$ astrocytoma with a calibrated score of 0.94 B. Detection of IDH1 mutation in glioma cell lines by sequencing analysis. C. Western blot of IDH1 mutant protein.

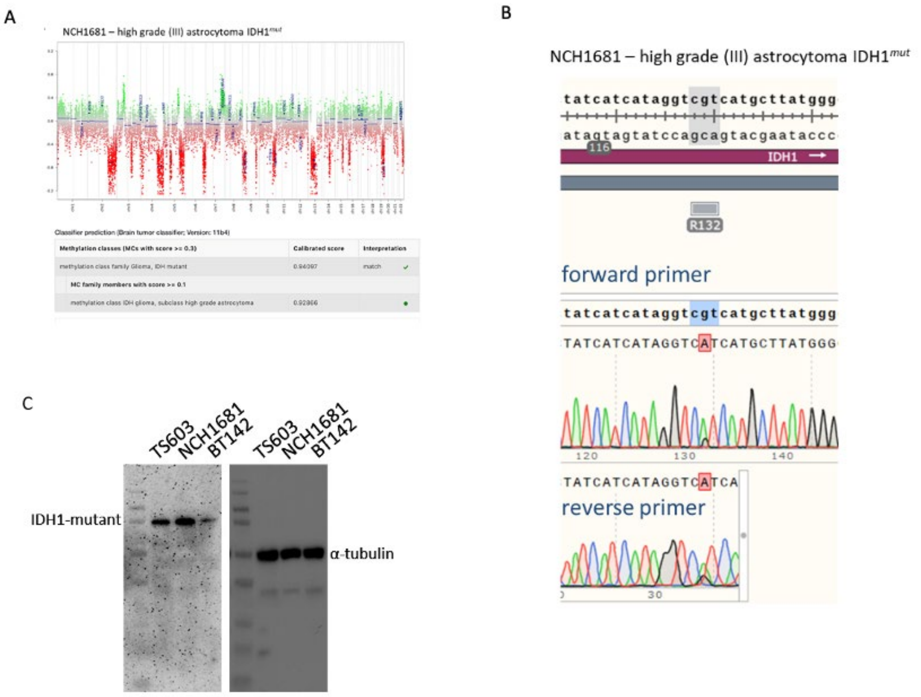

**Supplementary Figure S4**: **Radiotherapy modifies the metabolic profile of astrocytoma tissue**: **A**. Heat-map displaying the top 20 dysregulated metabolites in tumor tissue. **B.** Pathway analysis in which the main dysregulated metabolic pathways have been described.

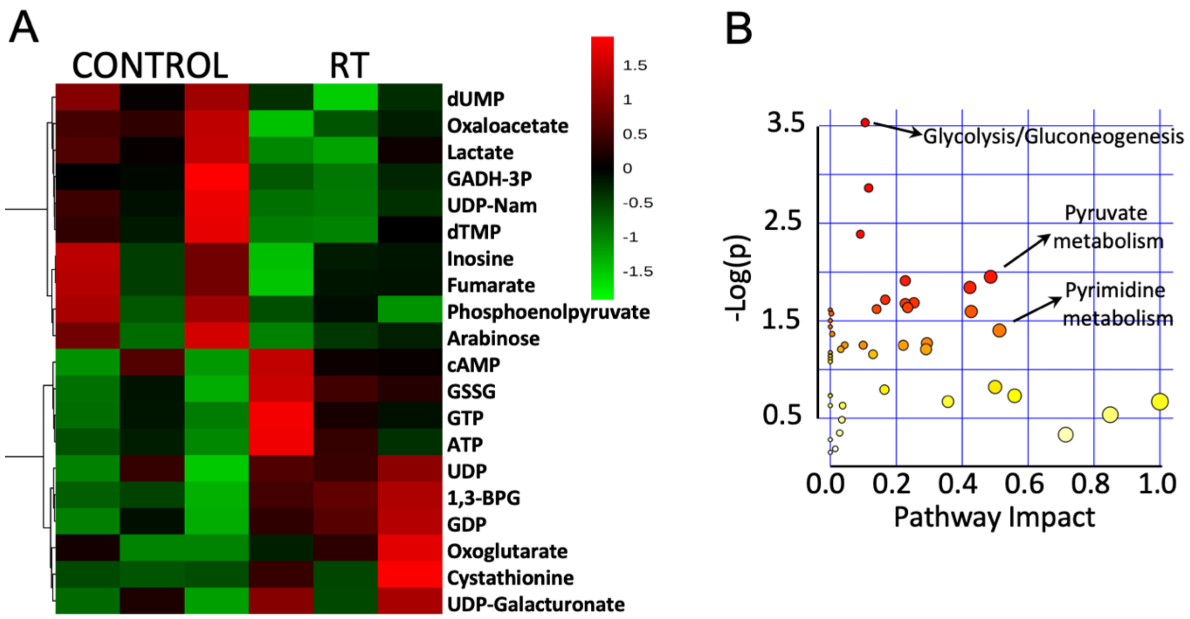

Supplement: Supplementary file 1 [file DataSheet_1.pdf]
